# Care provider interaction and psychological well-being of persons living with dementia in long-term care: a longitudinal observational study

**DOI:** 10.1186/s12912-023-01387-6

**Published:** 2023-06-28

**Authors:** Kyung Hee Lee, Eunjin Yang, Ji Yeon Lee

**Affiliations:** 1 Yonsei University College of Nursing and Mo-Im Kim Nursing Research Institute, Seoul, South Korea; 2 College of Nursing, Gachon University, Incheon, South Korea

**Keywords:** Communication, Dementia, Expressed emotion, Long-term care, Person-centered care, Social interaction

## Abstract

**Background:**

Although social interaction is important for dementia care and well-being of persons living with dementia, a limited number of studies have reported. This study aimed to examine whether the presence, type, and quality of social interaction is associated with psychological well-being among residents with dementia.

**Methods:**

This study analyzed 258 videos of 30 participants living with dementia. Social interaction was assessed by quality, type, and presence of interaction. Psychological well-being was measured by positive and negative emotional expressions. A mixed model was used for data analysis since these repeatedly measured observation data were nested within subjects.

**Results:**

Positive and neutral interactions were significantly associated with positive emotional expressions after controlling covariates, while negative interaction was significantly associated with negative emotional expressions. There was no significant relationship found between interaction presence or type and emotional expressions.

**Conclusions:**

This study showed interaction quality is essential to promote psychological well-being in persons living with dementia regardless of presence or type of interaction. This study highlights the importance of positive care provider interactions in dementia care. Additionally, institutional efforts to create an environment to reduce negative interactions appears essential to improve the psychological well-being of persons living with dementia.

**Trial registration:**

The study was reviewed and approved by the Yonsei University Institutional Review Board on October 16, 2020 (ref no: Y-2020-0158).

## Background

Across the Organisation for Economic Co-operation and Development (OECD) countries, approximately 10.7% of people aged 65 years and over received long-term care in 2019 [1], and this number is expected to grow with the change in demographics and ever-expanding aging population. Well-being is generally defined as “individuals’ subjective perceptions that life as a whole is good” [2: p. 331]. In the case of persons living with dementia, questions have been raised about whether their well-being is regarded with importance [3]. Unfortunately, these individuals are often denied basic rights and freedoms, such as through the use of restraints [4]. Nevertheless, well-being is an inherent right for all human beings, regardless of age or disease [3].

Since the conceptualization of personhood and well-being for persons living with dementia [5], concepts related to well-being have been widely applied and demonstrated, facilitating the measurement of the well-being of persons living with dementia regardless of the challenges [6]. Psychological well-being, which is a general feeling of wellness, is considered one of the subjective domains of quality of life [7]; in persons living with dementia, lower psychological well-being tends to be associated with lower cognitive impairment [8]. Although social interactions are an essential factor for improving well-being among nursing home residents [9], research has documented that social interaction between residents and nursing home staff is relatively low in long-term care facilities [10]. Moreover, social interaction with persons living with dementia is even more difficult [11] owing to their cognitive and physical impairments, as well as the behavioral and psychological symptoms in dementia.

Social interaction is not only a critical factor for the well-being of residents living with dementia but also an important goal of nursing home care [12] and person-centered dementia care. One of the person-centered care recommendations is to encourage purposeful and meaningful engagement [13], as this can be developed over time throughout the residents’ stay. In particular, the attitude of care providers and the quality of the social interaction between residents and care providers has an impact on the quality of life of residents in long-term care facilities [14]. Not all types of social interactions are constructive [15]; various forms of social interactions will cause different effects on individuals’ health and well-being based on their presence (i.e., interaction or no interaction), type (i.e., verbal, nonverbal, or both) and quality (i.e., positive, negative, or neutral). Since social interactions between care providers and residents usually occur when care providers are assisting with personal care, mealtimes, and activity programs, it is necessary to examine the type of social interaction, as well as the quality of the social interaction, that is related to well-being among persons living with dementia in long-term care.

There are, currently, only a limited number of studies that have reported findings on the relationship between social interactions and well-being in persons living with dementia [16, 17]. Specifically, a previous study pointed out that the presence of social interaction between residents and care providers is associated with higher positive affect in nursing home residents with dementia [16]; in another study, positive interaction (the quality of interaction) was associated with higher positive affect during daytime [15]. In terms of the type of interaction, using both verbal and nonverbal interactions might be more effective in improving the well-being of residents with dementia [17]. However, a research gap remains, as existing research has not examined the care-specific relationship between care providers and residents with dementia based on caregiving presence, type, and quality.

## Methods

### Aim

The purpose of this study is to examine the relationship between care provider-initiated interaction and psychological well-being among persons living with dementia in long-term care facilities. Specifically, we examined whether the presence (i.e., interaction vs. no interaction), type (i.e., verbal, nonverbal, and both interaction), and quality (i.e., positive, neutral, and negative interaction) of social interaction is associated with psychological well-being among persons living with dementia in long-term care.

### Study design and participants

This study conducted a secondary data analysis using data from a longitudinal observational study in which the emotional expressions of 30 persons living with dementia were examined. The participants resided in four different long-term care facilities. In the parent study, the optimum number of persons living with dementia was 24 (ANOVA repeated measured analysis with effect size = 0.25, α = 0.05, and power level = 0.80), and Cohen’s power analysis was performed to determine the study’s power. Considering the attrition rate, 30 persons living with dementia were recruited. The eligibility criteria for recruiting the participants were as follows: the participants had to (1) be 65 years of age or older, (2) be diagnosed with dementia using the Diagnostic and Statistical Manual of Mental Disorders, fourth edition, and (3) have a score of less than 24 points in a Korean Mini Mental State Examination (K-MMSE) [18]. The recruitment of care providers did not include any exclusion criteria, i.e., all care providers of all the persons with dementia were included. In the parent study, nine videos were taken of each participant at three intervals; at the start of the study, after three months, and after six months at three specific periods when care was provided, including mealtimes, personal care routines (e.g., washing of face, brushing of teeth), and leisure activities (e.g., recreation and exercise). Three participants dropped out of the study during the follow-up period because they were admitted to hospital (n = 2), and one participant passed away (n = 1), resulting in a total of 258 videos. Average length of video was 16.8 ± 12.1 min. A detailed description of the research design and methods is reported elsewhere [19].

### Variables and measures

#### Dependent variable

Psychological well-being was measured using the Philadelphia Geriatric Center Affect Rating Scale (PGCARS), which has been found to be a valid and reliable tool (kappa coefficients value = 0.76 to 0.89) to assess the emotional expressions of persons living with dementia [20]. Emotional expressions were used to measure psychological well-being because persons living with dementia find it difficult to verbally report their well-being, especially as the disease progresses, but they retain the ability to express emotions via facial expressions and body posture [20]. The original tool consisted of two domains, i.e., positive emotion and negative emotion. Pleasure, interest, and contentment were included in definition of positive emotional expression (PEE), whereas anger, anxiety, fear, and sadness were included in the definition of negative emotional expression (NEE). The corresponding emotional expression shown in the videos was counted for coding.

#### Independent variable

The quality, type, and presence of social interactions were assessed as independent variables. The interaction quality between care providers and the participants was measured using the Quality of Interaction Schedule (QUIS) [21]. This tool was initially used to measure the quality of social interactions between care providers and persons with mental illness. It showed good validity and reliability (kappa coefficients value = 0.60 to 0.91), and originally included five domains: positive social (e.g., greeting, chatting, conversation, offering choices, and encouragement), positive care (e.g., explanation and encouragement during delivery of care), neutral (i.e., a brief and indifferent interaction that is neither positive nor negative), negative protective (i.e., providing care without explanation or reassurance), and negative restrictive (e.g., ignoring and assaulting). In this study, the tool was modified to be suitable for video coding into three categories of positive interaction (i.e., positive social and positive care), neutral interaction, and negative interaction (i.e., negative protective and negative restrictive). The type of interaction that took place between care providers and the participants was coded using a coding scheme of verbal, nonverbal, and both (i.e., simultaneous verbal and nonverbal) interaction [17]. The no presence of interaction was also coded as no interaction when there existed a period of no interaction between care providers and participants in the videos.

### Covariates

The demographic and clinical data of the participants were gathered at baseline. These data included the age, sex and level of education of the participants, as well as the diseases they had been diagnosed with, the severity of their illness, and the medications they had been prescribed. To rate illness severity, the Cumulative Illness Rating Scale-Geriatric (CIRS-G) was used [22]. Diseases were categorized according to 14 systems of CIRS-G and then allocated 0–4 points according to their severity. The CIRS-G severity index (range 0–4) was determined by dividing the total score by the number of categories. In addition, information in respect of the medication prescribed to the participants was gathered and categorized into dementia, psychiatric, cardiovascular, diabetes, and other medications. Information regarding the four facilities (e.g., ratio of care providers to residents and number of beds) was gathered at baseline.

Data collected at three specified time periods included information on the participants’ cognition, level of depression, and functional status, as well as the care providers who provided services during each of the nine care events. Participants’ cognition was evaluated using both the K-MMSE, in which all participants had a score of 24 points or less, and the Korean version of Clinical Dementia Rating (K-CDR) [18, 23]. The K-MMSE, a 30-item tool, indicates a decrease in cognitive function with a lower score. The scores of the participants ranged from − 1 to 30; where the K-MMSE was not testable the participant was given a score of -1 [18]. The K-CDR, a 6-item tool (range 0–18), indicates a decrease in cognitive function with a higher score [23]. Both are globally validated tools for assessing cognitive impairment. Participants’ depression was evaluated by the Korean version of the Cornell Scale for Depression in Dementia (K-CSDD). This 19-item tool (range 0–38) has also been assessed to be a valid and reliable tool in evaluating the risk of depression in persons living with dementia [24, 25]. A higher score denotes a higher level of depression. Participants’ functional status was measured using the Korean version of Activities of Daily Living (K-ADL) [26, 27]. This 7-item tool (range 7–21) has also been recognized as a valid and reliable tool in assessing functional capacity, and increased dependency was noted in a higher score in K-ADL.

Care providers consisted of individuals from various professions, including nurses, caregivers (i.e., *Yo-yang Bohosa* in South Korea, who have a similar role to nursing aids in a Western country), social workers, physical therapists, occupational therapists, instructors, music therapists, volunteers, and family members. Care providers were categorized into nursing care providers (i.e., nurses and caregivers [*Yo-yang Bohosa*]), non-nursing care providers (i.e., therapists, social workers, and instructors), and others (i.e., volunteers and family members). Depending on the type of care services delivered, professions providing care were generally determined in long-term care. Most mealtime and personal care services were provided by nursing care providers, whereas most leisure activity services were provided by non-nursing care providers.

### Procedure and coding

In the parent study, written consent was obtained from legal proxies, and verbal assent was obtained from persons living with dementia before every video recording session. Care providers who appeared in the videos were informed of the study and gave written consent to being observed and recorded by a camera. The camera was installed such that it did not intervene with the interaction between care providers and persons living with dementia during service provision. Research assistants (RAs) who filmed the videos received training on ethical issues such as privacy. In this study, the secondary analysis was reviewed by the relevant Institutional Review Board (IRB). After the IRB approval, 258 videos were extracted from the parent study. Before the commencement of coding, three coders (RAs) were intensively trained in video coding, the protection of the privacy of the participants, ensuring confidentiality, preventing the release of the videos, and research ethics. Initially intra-rate/inter-rater reliability for interaction coding was examined using the same videos between coders. After confirming the high kappa coefficient value (0.9–0.95), coding began. To assess the reliability of the coding, at least two RAs coded the same video, and thereafter one RA coded each video. We monitored the reliability throughout the course of the study (before, in the middle, and at the end of coding) and maintained high kappa values (up to 1.0).

The coding was conducted using Noldus Observer® XT software as follows: videos were imported into the software. Coding schemes were pre-embedded in the software for social interaction quality and type according to the categories mentioned in the above tool (e.g., QUIS), and for emotional expression using the PGCARS. The video stopped playing after 10 s and the coder decided which type or quality of interaction appeared in the video. If there was no interaction between care providers and the participants, it was coded as no interaction. In the case of emotion coding, the video paused after every five seconds of play as emotional expressions change more rapidly than interactions. If the coders had difficulty in deciding which categories of interaction or emotional expressions corresponded with what they had seen, a research meeting was held and the part of the video in question was identified, replayed, and discussed. Coders took breaks between viewing the videos, and the maximum number of hours they spent video coding was limited to three hours per session to prevent coders’ fatigue.

### Data analysis

Since the total video length was different for each video, coding data values were standardized per minute for analysis. To indicate how many times each emotion appeared per minute (PEE/NEE rate), the total number of times (frequency) of each displayed emotion was divided by the video length (minutes), generating relative frequency (frequency per minute). Interaction data were also standardized into the form of frequency per minute. A mixed model was chosen for the data analysis performed in this study. Mixed model analysis involves the repeated measurement of longitudinal data when the data have a multi-level structure [28]. During each service, three videos were taken repeatedly at three time points per individual residing in four facilities; thus, the data were nested within subjects in a hierarchical structure.

All statistical analysis was performed using the STATA 16.0 software (StataCorp, College Station, Texas, USA) according to the following process. First, we conducted a descriptive analysis to explore the mean, variance, and percentage of each variable. Thereafter, all analyses were conducted for each of the dependent variables, i.e., PEE rate and NEE rate. The distribution of each dependent variable was different. PEE rate had right-skewness and the mean was closely clustered to the highest value (mean value = 10.10; highest value = 12). Therefore, a multilevel mixed-effect Tobit regression was used for the analysis of the PEE rate. The NEE rate, on the other hand, had left-skewness, and the variance (i.e., 7.23) was significantly larger than the mean value (i.e., 1.72). Therefore, a multilevel mixed-effects generalized linear model where the dependent variable was set as a negative binomial distribution was used.

Second, the mixed model analysis was conducted for the bivariate analysis to identify variables that have an association with each dependent variable. Variables that showed statistical significance in the bivariate analysis were chosen as covariates in the final analysis. These variables were K-MMSE, CDR, ADL, care providers, care services, and facility in case of PEE rate and K-MMSE, CDR, ADL, taking dementia medications, care providers, care services, and facility in case of NEE rate.

Finally, for each dependent variable (i.e., PEE and NEE rate), the final analysis was conducted using the independent variable (i.e., interaction), covariates (i.e., CDR, ADL, care providers, and facility in the PEE model, and the former plus taking dementia medication in the NEE model), and time as fixed effects, as well as individual subject as a random effect. Although the facility was included as a variable in the final model, the results displayed were suppressed due to confidentiality issues. The type of care services was not included in the final analysis since a certain service was provided by a certain profession and this would lead to multicollinearity with care providers. For the same reason, the final analysis only included CDR scores instead of K-MMSE scores, as most of the participants had low scores.

## Results

The characteristics of the participants are shown in Table 1. A total of 28 female and two male participants whose mean age was 85.63 years (standard deviation [SD] = 6.67) took part in the study. The CIRS-G severity index shows their illness severity was 1.75 (SD = 0.70) and the number of dementia medications they were taking was 1.70 (SD = 0.94). The mean score was 16.42 (SD = 2.24) in CDR, 3.72 (SD = 3.44) in CSDD, and 18.51 (SD = 2.21) in ADL. The mean PEE rate was 10.10 (SD = 2.74) frequency per minute, indicating a more frequent repetition than the NEE rate (mean = 1.72, SD = 1.69).

**Table 1:** Characteristics of participants (n = 30)

Characteristics	Mean (SD)	n (%)
Age	85.63 (6.67)	
Sex		
Female		28 (93.30)
Level of education		
< Middle school		16 (53.30)
≥ Middle school		14 (46.67)
CIRS-G severity index^a^	1.75 (0.70)	
Number of dementia medications	1.70 (0.94)	
K-MMSE score^b^	2.81 (5.18)	
CDR score^b^	16.42 (2.24)	
CSDD score^b^	3.72 (3.44)	
ADL score^b^	18.51 (2.21)	
Emotional expression^c^		
PEE	10.10 (2.74)^d^	
NEE	1.72 (2.69)^d^	

*Notes*. ADL = Activities of Daily Living; CDR = Clinical Dementia Rating; CIRS-G = Cumulative Illness Rating Scale-Geriatric; CSDD = The Cornell Scale for Depression in Dementia; K-MMSE = Korean Mini Mental State Examination; NEE = Negative Emotional Expression; PEE = Positive Emotional Expression; SD = Standard Deviation

^a^ Total CIRS-G score/total number of categories endorsed

^b^ n = 86

^c^ n = 258

^d^ frequency per minute

Table 2 sets out the relative frequency of each interaction, viz., interaction presence, interaction quality, and interaction type. The mean of no interaction was 4.37 ± 2.58, while that of interaction presence was 1.62 ± 1.89. As for the interaction quality, the mean of positive interaction was 0.71 ± 1.08, that of neutral interaction was 0.70 ± 1.41, and that of negative interaction was 0.21 ± 0.64. Finally, as for the interaction type, the mean of nonverbal interaction was 0.85 ± 1.50, that of verbal interaction was 0.57 ± 0.92, and that of verbal interaction was 0.23 ± 0.47.

**Table 2:** Relative frequency of interaction according to the presence, quality, and type (n = 258)

Interaction	Mean (SD)^a^
Presence	
No interaction	4.37 (2.58)
Yes interaction	1.62 (1.89)
Quality	
Positive interaction	0.71 (1.08)
Neutral interaction	0.70 (1.41)
Negative interaction	0.21 (0.64)
Type	
Verbal interaction	0.23 (0.47)
Nonverbal interaction	0.85 (1.50)
Both interaction	0.57 (0.92)

*Notes*. n = number of videos

^a^ Frequency per minute

The associations between each interaction and emotional expression (PEE/NEE rate) are displayed in Table 3. With the covariates controlled, the PEE rate was found to be statistically significant with both positive and neutral interactions (*β* = 3.31, *p* = .049; *β* = 3.98, *p* = .028, respectively). That is, the more positive and neutral the care providers’ interactions were with the participants, the more positive emotions were expressed by the participants. In this PEE model, fewer positive expressions were presented when the non-nursing care providers (*β* = -14.26, *p* < .001) were involved in the care event instead of nursing care providers. In the case of NEE, its rate was found to be statistically significant with negative interaction (*β* = 2.49, *p* = .021). Specifically, the more negative the care providers’ interactions were with the participants, the more negative emotions were expressed by the participants. In this NEE model, fewer negative expressions were presented when non-nursing care providers (*β* = -7.30, *p* < .001) and volunteers and family members (*β* = -6.12, *p* < .001), rather than nursing care providers, were involved in the care event.

**Table 3:** Association between interaction and emotional expression (n = 258)

			Positive Emotional Expression^a^			Negative Emotional Expression^a^		
Variables			Coefficient	SE	*P*-Value	Coefficient	SE	*P*-Value
Interaction								
	No interaction		0.49	0.34	0.152	0.02	0.11	0.888
	Quality	Positive	3.31	1.68	0.049	0.59	0.98	0.549
		Neutral	3.98	1.82	0.028	1.40	1.13	0.213
		Negative	2.61	1.94	0.179	2.49	1.08	0.021
	Type	Verbal	1.86	1.65	0.259	1.27	0.99	0.201
		Nonverbal	-2.19	1.76	0.214	-0.25	1.08	0.819
		Both	-2.90	1.89	0.125	1.27	1.13	0.262
CDR			-0.24	0.47	0.606	0.28	0.26	0.287
ADL			-0.24	0.50	0.627	-0.19	0.35	0.597
Taking dementia medication								
	Yes		—			-1.79	3.97	0.652
Care providers								
	Nursing		Reference					
	Non-nursing		-14.26	1.54	< 0.001	-7.30	1.46	< 0.001
	Others		-5.02	2.58	0.052	-6.12	1.55	< 0.001
Time			0.10	0.72	0.886	-0.50	0.40	0.205

*Notes*. n = number of videos

ADL = Activities of Daily Living; CDR = Clinical Dementia Rating; SE = Standard Error

^a^Analysis included variables for four facilities to control for their effects, but result display was suppressed

## Discussion

This study investigated the relationship of the social interactions between care providers and persons living with dementia, as well as the emotional expressions of persons living with dementia during long-term care. It was discovered that positive and neutral interactions were related to PEEs, while negative interactions were related to NEEs. To our knowledge, this is the first quantitative study making use of video tape recordings to reveal such a relationship in a care-specific context across care providers. The results of the current study are significant in some respects.

We found that positive/neutral care provider-initiated interaction was related to PEEs, while negative interaction was related to NEEs, on the condition of controlling for covariates such as characteristics of care recipients, providers, and facilities in care events. These results corroborate the findings of many previous studies that identified quality interaction is likely to be associated with positive emotion [16, 17]. However, further questions in respect of the relationship and its extent (i.e., how deep the relationship is) between care provider-initiated interaction and specific emotions among persons living with dementia in a care context remain unanswered. The most obvious finding to emerge from our analysis was that there was a clear correlation between interaction quality and emotional expression and that the coefficient value of the PEE model was 3.31 and 3.98 in positive and neutral interactions, respectively. Positive interaction includes care providers’ attempts to empathize and understand the participant by addressing their needs, which eventually made them feel valued, supported, and empowered, thus encouraging them to interact more [29]. When persons living with dementia are kept in good spirits, they are more likely to maintain their social interactions and stay connected to others.

In addition, we attempted to identify what types of interaction are related to the participants’ emotions, and we found out that interaction quality matters regardless of the type or quantity of interaction. The care situation, especially the long-term one, provides the opportunity for both care providers and residents to interact. We believe that residents should be encouraged to participate purposefully in activities, express their preferences about food or lifestyle, and make their own choices during care situations. When a good emotional expression is found among persons living with dementia, they can be engaged in social activities, and this encourages further social interaction. Clinically, this finding is significant as it supports prior evidence that interaction quality matters when applying person-centered care.

Regarding negative interaction, however, we found that not all care provider-initiated interactions are positive. This also accords with earlier observations, which showed that there are task-centered care provider behaviors that are related to behavioral symptoms (e.g., agitation) or resistiveness to care among persons living with dementia [30, 31]. Fortunately, there is room for improvement in the scope of interaction initiated by care providers through educational or institutional efforts. Previous studies reported that the role of care providers is an important environmental factor in improving interaction in a reality where persons living in long-term care settings have less social interaction [11, 32]. There might be a change in the relationships of persons living with dementia in long-term care facilities in that they have less contact with their family and friends due to their physical location. In such an environment, the care providers’ role is important to help persons living with dementia to form positive reciprocal relationships with others [33]. To achieve this, fostering a caring philosophy or care approach that facilitates positive interaction is vital because it can influence the way care providers interact with persons living with dementia.

Another vital clinically relevant finding was that nursing care providers’ interactions were more related to positive emotions, while interactions with non-nursing care providers and other individuals were related to less negative emotions. This might be explained in that certain care providers were closely involved with certain types of care situations. For example, the personal care situation, which can be a challenging time for care providers as many residents dislike these routines, was provided by nursing care providers. Likewise, mealtimes, which are used to relieve hunger, can be a challenging time when residents resist eating [30, 31]. In addition, the nursing care provider group is the main caregiving group, and the time this group spends interacting with residents is much longer than that of other providers. On the other hand, non-nursing care providers are usually associated with leisure activities that persons living with dementia might show an interest in, and thus, this can encourage residents to engage in the activities and may improve their social interactions. Therefore, it is important to provide appropriate clinical guidelines to enhance the social interactions according to the providers’ type and the services provided. Regarding cognition, whether positive or negative, cognitive function was not found to be related to emotional expression. A possible explanation for this might be that our study population was a highly cognitively impaired group, most of whom had severe dementia and whose mean K-MMSE score was 2.8. Despite this, as have others [16], there was a tendency to show more positive emotion with a lower score in CDR (i.e., good cognitive function).

This study provided more in-depth knowledge about social interactions between care providers and residents living with dementia. Even during care, no interaction occurred 2.70 times more than interaction. Although it has already been established that social interactions were low in long-term care settings from previous studies [9], we have provided empirical data regarding low interaction rates even during care. A possible explanation for this might be that long-term care providers are focused on finishing the tasks they are assigned instead of interacting in a meaningful manner with the persons under their care. Although social interactions were inadequate during care, it is encouraging to note that there were more positive interactions than negative interactions on the whole. To improve care quality, it is recommended that care providers spend more time offering positive interaction during care. Additionally, long-term care providers mainly use nonverbal or both nonverbal and verbal interactions instead of verbal interactions solely. Since dementia-related language function declines in the fairly early stages [34], care providers should be encouraged to include nonverbal interaction methods to promote interaction with persons living with dementia. A systematic review also suggested nonverbal communication could be utilized to supplement verbal communication to improve communication skills, and thus the social interactions which take place between caregivers and residents [35].

Our study has several limitations. Although this was a six-month follow-up study, our results only showed a correlation, not a causal relationship between care provider-resident interactions and residents’ emotional expressions. In addition, although the minimum number of participants needed to obtain sufficient statistical power was met, the small sample size limited the generalizability of the results. As such, future studies with a large sample, using sequential analysis designed to clarify the causal relationship between care provider-resident interactions and responses, will need to be carried out. Nevertheless, the strengths of this study included that video observations, as objective and reliable measures that compare to self- and proxy-reported, were used, and this produced reliable results through a mixed model analysis — considering the characteristics of the data. Additionally, the six-month study period and accompanying lapse in time allowed us to capture more natural interactions between the care providers and the participants.

## Conclusions

This study revealed the relationship between social interaction of care providers and persons living with dementia and the emotional expression of persons living with dementia. Our results highlight the importance of positive care provider-initiated interactions in dementia care. This study has significant implications for long-term care staff and administrators of care institutions to ensure that care providers interact positively with persons living with dementia when providing care. Efforts need to be made at an institutional level to create an environment that reduces negative interactions between caregivers and residents, as this can be of great assistance in promoting the psychological well-being of persons living with dementia.

## Data Availability

The datasets utilized for this study are not publicly available due to IRB agreements; however, they are available from the corresponding author on reasonable request.

## List of abbreviations

CIRS-G: Cumulative Illness Rating Scale-Geriatric
IRB: Institutional Review Board
K-ADL: Korean Version of Activities of Daily Living
K-CDR: Korean Version of Clinical Dementia Rating
K-CSDD: Korean Version of Cornell Scale for Depression in Dementia
K-MMSE: Korean Mini Mental State Examination
NEE: Negative Emotional Expression
OECD: Organisation for Economic Co-operation and Development
PEE: Positive Emotional Expression
PGCARS: Philadelphia Geriatric Center Affect Rating Scale
QUIS: Quality of Interaction Schedule
RA: Research Assistant
SD: Standard Deviation
